# Epidemiological Trends and Age–Period–Cohort Effects on Dengue Incidence Across High-Risk Regions from 1992 to 2021

**DOI:** 10.3390/tropicalmed10060173

**Published:** 2025-06-18

**Authors:** Yu Cao, Hanwu Chen, Hao Wu, Bin Wu, Lu Wang, Xin Liu, Yuyue Yang, Hui Tan, Wei Gao

**Affiliations:** 1 School of Public Health, Jiangxi Medical College, Nanchang University, 461 Ba Yi Avenue, Nanchang 330006, China; 356500230005@email.ncu.edu.cn (Y.C.); chenhanwu2023@163.com (H.C.); w648308194@163.com (H.W.); wubin8991@163.com (B.W.); wanglu07080817@163.com (L.W.); 406500240012@email.ncu.edu.cn (X.L.); 406500240018@email.ncu.edu.cn (Y.Y.); 416500240050@email.ncu.edu.cn (H.T.); 2 Jiangxi Provincial Key Laboratory of Disease Prevention and Public Health, Nanchang University, 461 Ba Yi Avenue, Nanchang 330006, China

**Keywords:** dengue, incidence, temporal trends, high-risk areas, age–period–cohort analysis

## Abstract

Dengue, an acute infectious disease caused by the dengue virus, remains a major public health problem in the 21st century. This study investigated the global dengue burden, identified high-risk regions, evaluated the long-term incidence trends, and can inform evidence-based control strategies. Using GBD 2021 data, we analysed the dengue incidence from 1992 to 2021 using age–period–cohort models. We determined the net drift (overall annual percentage change), local drift (annual percentage change for each age group), longitudinal age curves (expected longitudinal age-specific rates), and periods’ (cohorts’) relative risks. In 2021, the global age-standardised incidence rate reached 752.04/100,000 (95% UI: 196.33–1363.35), a 47.26% increase since 1992. High-risk regions included eastern sub-Saharan Africa, Southeast Asia, South Asia, and Latin America and the Caribbean. Southeast Asia experienced the largest rise (65.43%), with a net drift of 2.47% (1992–2021). While individuals aged 5–39 years bore the highest burden, those over 80 faced an elevated risk. Dengue remains a critical public health threat, disproportionately affecting younger populations but increasingly endangering older adults. Targeted interventions in high-risk regions and age groups, coupled with precision public health strategies, are essential to enhance prevention and control efforts.

## 1. Introduction

Dengue is a viral illness of acute onset, predominantly spread by bites from infected mosquitoes, especially *Aedes aegypti* [1,2]. Infection with any dengue virus serotype can lead to symptoms such as fever, nausea, vomiting, rashes, and pain. In severe cases, patients may develop haemorrhage and shock, with an untreated mortality rate reaching up to 20% [3]. Over the past three decades, the reported global incidence of dengue has escalated substantially, rising from 30.7 million cases in 1990 to 56.9 million in 2019 [4]. The disease is now endemic in over 100 countries across tropical and subtropical regions, primarily affecting low- and middle-income nations [5,6]. Projections indicate that by 2080, the share of the world’s population exposed to dengue risk may have risen from 53% in 2015 to 63% [7]. In endemic areas, particularly in Southeast Asia, dengue predominantly affects children, with case fatality rates ranging between 3% and 10% [8]. The global healthcare burden of dengue exceeds USD 8.9 billion annually [9,10]. In Southeast Asia alone, the economic cost between 2001 and 2010 was USD 950 million per year, with a corresponding yearly loss of 372 disability-adjusted life years per million residents [11]. Outbreaks not only put enormous strain on health systems but also undermine public confidence in governmental responses.

Dengue is mainly concentrated in tropical and subtropical regions, which are usually characterised by dense populations, rapid population movements, limited healthcare infrastructure, a high concentration of low- and middle-income countries, and environmental conditions conducive to the reproduction of mosquitos, dengue’s main vectors, making them particularly susceptible to dengue outbreaks. To address this significant public health threat, national governments and the World Health Organization (WHO) have implemented a range of integrated control strategies. These include traditional vector control measures—such as insecticide spraying, the elimination of larval habitats, and indoor residual spraying—as well as public health education campaigns, disease surveillance systems, and, more recently, targeted vaccination programmes [12,13,14]. However, the effectiveness of these interventions has varied substantially across regions [15]. Therefore, it is essential to assess and compare the dengue incidence trends in high-risk regions to inform resource allocation and improve the design and delivery of intervention strategies.

While recent advancements in global disease burden studies have provided systematic estimates of dengue’s health impact, the existing frameworks predominantly focus on describing the current burden of dengue, leaving knowledge gaps in our understanding of long-term epidemiological transitions and their underlying drivers in high-risk endemic regions [16,17]. As a vector-borne disease, dengue’s transmission dynamics cannot be fully understood without considering age-specific susceptibility, spatiotemporal transmission patterns, cohort-specific exposure profiles, changes in disease surveillance capacities, and the implementation of preventive and control measures. The age–period–cohort (APC) model offers a valuable analytical framework to elucidate the relationship between the dengue incidence trends and epidemiological effects in high-risk areas. Such insights are crucial for identifying the macro-determinants of disease risk, evaluating the effectiveness of health policies, and informing targeted strategies to improve dengue management. In addition, with the COVID-19 pandemic disrupting health systems and human mobility patterns between 2020 and 2022, it is necessary to examine whether this has influenced the dengue incidence trends in high-risk areas.

Using GBD 2021 data, this study analysed the global trends in the dengue incidence by age, sex, time periods, and geographic regions from 1992 to 2021, with a particular focus on high-risk regions. The analysis provides updated insights into the epidemiology of dengue in high-risk regions, expanding upon prior research. Furthermore, the APC model was employed to assess how age, the period, and the birth cohort have influenced shifts in the dengue incidence over the past 30 years, exploring the mechanisms underlying these temporal variations. Through the application of the APC framework to high-risk regions, this research elucidates spatial and temporal patterns in the dengue incidence and offers valuable evidence to inform tailored intervention and control efforts globally.

## 2. Materials and Methods

### 2.1. Data Source

Data on populations and the dengue incidence were extracted from the GBD 2021 database using the Global Health Data Exchange query tool “http://ghdx.healthdata.org/gbd-results-tool (accessed on 15 January 2025)”. The GBD 2021 study provided yearly estimates of the incidence and mortality for 371 diseases and injuries across 204 countries and territories spanning the years from 1990 to 2021. The detailed methodology for the GBD 2021 is available elsewhere [18,19]. In the GBD2021, dengue was defined according to the International Classification of Diseases, 10th edition (ICD-10), incorporating categories A90 (classical dengue fever) and A91 (dengue haemorrhagic fever) [19]. The GBD database presents estimates as the age-standardised rates per 100,000 individuals. Each estimate is accompanied by a 95% uncertainty interval (UI), which is derived by replicating the sample 500 times, with the upper and lower bounds based on the 2.5th and 97.5th percentiles of the uncertainty distributions [18]. The relevant data were anonymized and publicly available, and the University of Washington Institutional Review Board reviewed and approved the waiver of informed consent.

### 2.2. Data Collection and Processing

We report the global dengue incidence rates and their distribution patterns, with a particular focus on four high-risk dengue-endemic regions: eastern sub-Saharan Africa (15 countries), South Asia (5 countries), Southeast Asia (13 countries), and Latin America and the Caribbean (33 countries). Supplementary Table S1 shows the composition of the countries. Within high-risk regions, countries that had successfully eliminated dengue by 2021 were excluded from the regional analyses. In this study, we obtained population data, dengue case counts, crude incidence rates, and age-standardised incidence rates (ASIRs) for four high-risk regions and countries within the regions for the years of 1992–2021, with ages classified into 19 groups ranging from 0–4 years to 90–94 years.

### 2.3. Descriptive Analysis

This study reports on the global incidence of dengue and its spatial and temporal trends from 1992 to 2021. Based on visualised maps, we illustrated the spatial distribution of the dengue incidence across 204 countries and territories in 1992 and 2021. To assess temporal trends, we analysed age-specific incidence rates, ASIRs, and relative percentage changes in the incidence over the entire study period (1992–2021) and the recent period (2019–2021).

### 2.4. Age–Period–Cohort Model of Dengue Incidence

This study employed the APC modelling framework to examine underlying trends in the dengue incidence across different age groups, time periods, and birth cohorts, aiming to explore how macro-level factors influence the disease risk. Specifically, the age effect captures variations in the disease risk across the lifespan, attributable to biological ageing, cumulative exposure, or changing susceptibility. The period effect reflects temporal changes in the incidence driven by factors such as the availability of screening, disease outbreaks, or public health interventions during specific time periods. The cohort effect represents generational differences in the risk, shaped by distinct exposure patterns or behaviours associated with particular birth cohorts [20,21]. Typically, the APC model fits a log-linear Poisson regression to incidence data presented in a Lexis diagram and quantifies the additive contributions of age, the period, and the cohort. However, since age, the period, and the cohort are linearly dependent (cohort = period − age), the model faces an “identification problem”, whereby the unique estimation of all three effects is statistically impossible [19]. This limitation may be addressed using the intrinsic estimator (IE) approach, which introduces constraints that allow for the derivation of identifiable functions and meaningful temporal trends [22].

The APC model requires alignment in the interval structure of age and period groups. As the GBD estimates are produced in an irregular data format—specifically, annual data structured in five-year age groups—the estimated dengue incidence rates from the GBD 2021, along with population data for each country or region, were used as the input for the APC model. For this analysis, dengue data were stratified based on the following rules. Age groups: Nineteen five-year age categories ranging from 0–4 to 90–94 years. Period groups: Six consecutive 5-year periods from 1992–1996 (average of 5 years) to 2017–2021 (average of 5 years). Birth cohort group: A total of 24 birth cohorts calculated based on the age–period matrix, spanning from 1898–1906 to 2013–2021. Supplementary Table S2 presents a Lexis diagram of the dengue dataset used in the APC model, showing the corresponding age, period, and cohort combinations [23].

We evaluated several key APC parameters: the net drift, denoting the overall annual percentage change in the ASIRs; the local drift, indicating age-specific annual rate changes; longitudinal age curves reflecting the longitudinal age-specific incidence rates within the reference cohort, adjusted for period effects; and the period (or cohort) relative risk (RR), which represents the different period (or cohort) RR, adjusted for age and cohort (or period) effects [21]. Within the APC model, the reference categories for RR estimation were the 2002–2006 period and the 1978–1986 birth cohort, with RR values exceeding (below) 1 signal higher (lower) than the dengue incidence risk. The statistical significance of the estimable parameters and derived functions was assessed using the two-sided Wald chi-squared test. The APC analyses were conducted using the online APC network tool “https://analysistools.cancer.gov/apc/ (accessed on 11 March 2025)”. Data visualisation was performed using R statistical software (version 4.4.0).

## 3. Results

### 3.1. Global Distribution of Dengue Incidence, 1992–2021

**Figure 1:**
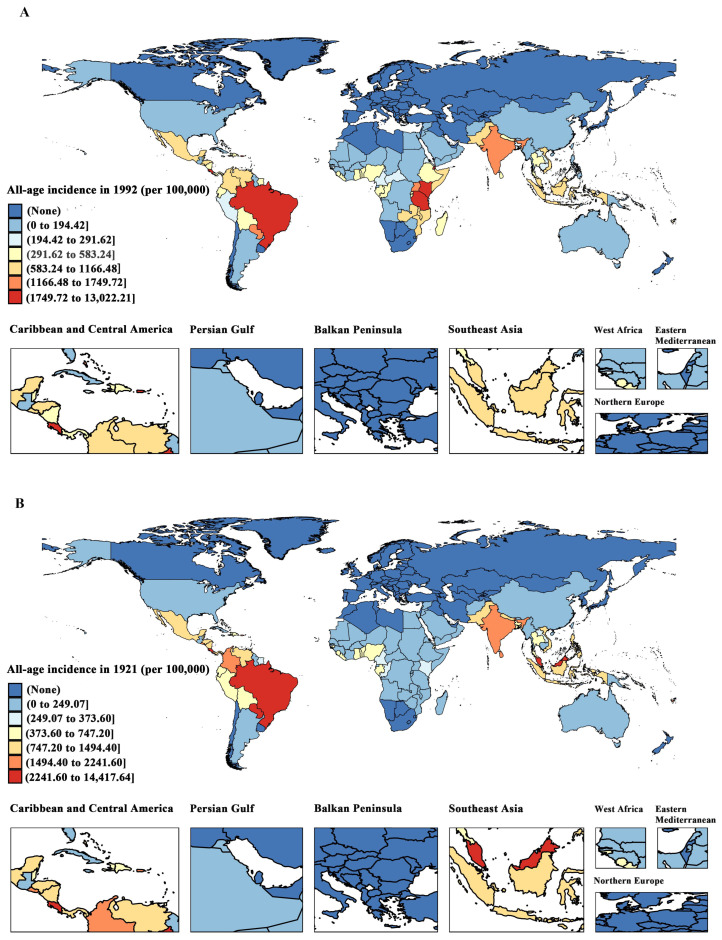
The crude incidence of dengue across 204 countries and territories in the years 1992 (**A**) and 2021 (**B**).

Figure 1 illustrates the geographical distribution of the dengue incidence across 204 countries from 1992 to 2021. In 1992, the high-incidence areas were mainly in eastern sub-Saharan Africa, South Asia, Southeast Asia, and Latin America and the Caribbean. By 2021, these areas had shifted to Southeast Asia, Latin America and the Caribbean, and South Asia. Globally, dengue incidents have risen to 589.64/100,000, with an incidence rate of 747.2/100,000. However, in eastern sub-Saharan Africa, the incidence rate has decreased to 90.97/100,000.

### 3.2. Trends in Dengue Incidence in High-Risk Areas, 1992–2021

Table 1 summarises the data on the population size, total number of dengue cases, crude incidence, ASIRs, and net drift in ASIRs across global regions. The global dengue incidence increased markedly between 1992 and 2021, with an average of 589.64/100,000. In particular, dengue cases have seen a significant rise in South and Southeast Asia, as well as in Latin America and the Caribbean. South Asia experienced the most marked escalation, where the incidence rate surged to 318.12/100,000, reflecting a rise of 137.26%. Over the study period, ASIRs exhibited an upward trend globally, as well as in all regions except eastern sub-Saharan Africa. Southeast Asia experienced the most dramatic increase, with a rise of 65.43%, reaching an ASIR of 971.89/100,000. Results from the APC analysis suggest a general upward trend in the global dengue incidence. However, the incidence in eastern sub-Saharan Africa declined, with an average annual decrease of 9.02% (95% UI: −9.07 to −8.98).

During the COVID-19 pandemic (2019–2021), both the increases and decreases in the dengue incidence and ASIRs in South Asia, Southeast Asia, and Latin America and the Caribbean slowed markedly, indicating a period of relative stability. The temporal patterns of the dengue incidence across countries in high-risk regions from 1992 to 2021 are detailed in Supplementary Table S3.

### 3.3. Temporal Changes in Age-Specific Dengue Incidence in High-Risk Areas, 1992–2021

**Figure 2:**
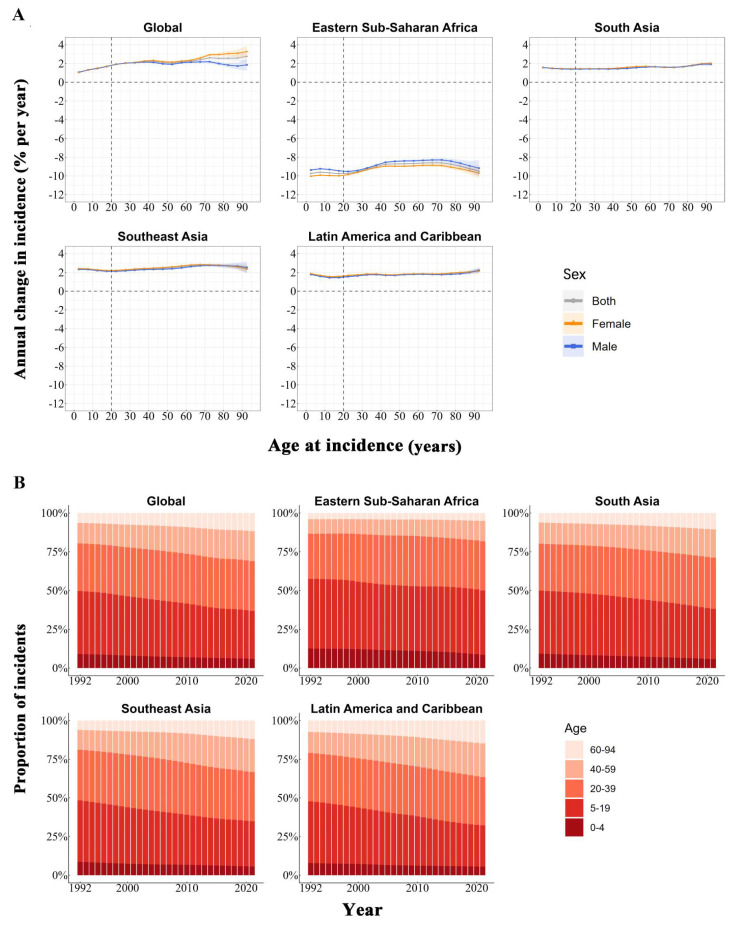
Temporal dynamics of dengue incidence by age group in high-risk regions, 1992–2021: (**A**) Estimated local drift values of dengue incidence from age–period–cohort analysis across 19 age categories (spanning from 0–4 to 90–94 years) during 1992–2021. (**B**) Changes in age-specific distribution of dengue cases across age groups (0 to 4, 5 to 19, 20 to 39, 40 to 59, and 60 to 94 years), 1992–2021.

Figure 2A displays the local drift estimates, reflecting the yearly rate of change in the dengue incidence across successive 5-year age intervals spanning from 0 to 94 years. From 1992 to 2021, positive local drift values across all the age bands globally indicated a continuous rise in the dengue incidence. In contrast, eastern sub-Saharan Africa exhibited negative values throughout, suggesting a sustained decline over the same period. No notable sex-specific differences in the local drift estimates were observed across the studied regions. The country-specific local drift results for the four high-risk regions are shown in Supplementary Figures S3 and S4.

Figure 2B shows the temporal trend in the proportion of dengue cases across different age groups. Globally, the 5–19 and 20–39 age groups were the most affected. Consistent with the global patterns, the 5–19 and 20–39 age groups exhibited the greatest number of dengue cases across all four high-risk regions. Supplementary Figures S3 and S4 illustrate the age- and sex-specific distribution of dengue cases across the four high-risk regions from 1992 to 2021.

### 3.4. Age, Period, and Cohort Effects on Dengue Incidence Across High-Risk Regions, 1992–2021

**Figure 3:**
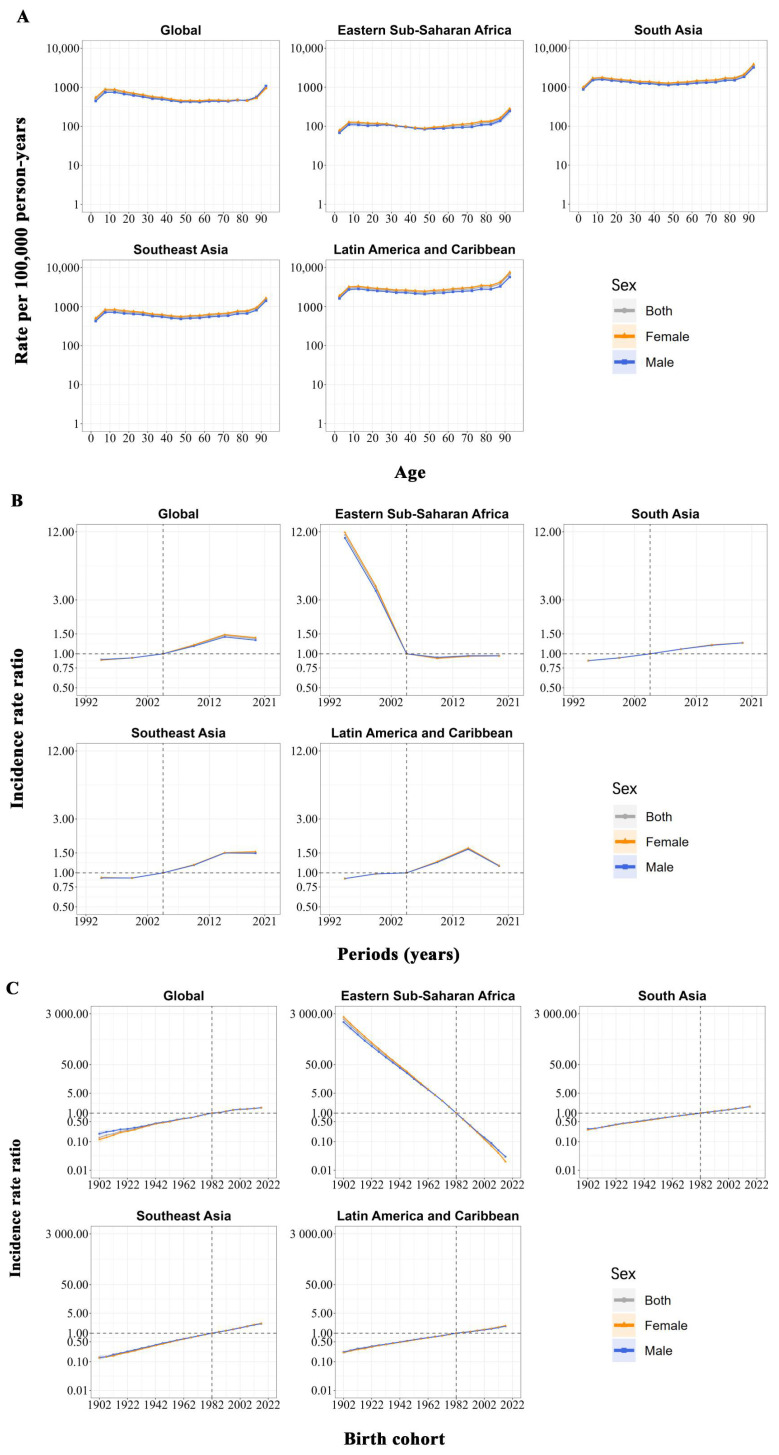
Age, period, and cohort effects on dengue incidence across high-risk regions. (**A**) Age effects represent age-specific trends in incidence rates adjusted for temporal variations. (**B**) Period effects are expressed as relative risk of incidence, calculated by comparing age-specific incidence rates during each period to those observed in reference period (2002–2006). (**C**) Cohort effects are presented as relative risks of incidence, calculated by comparing age-specific incidence rates across birth cohorts (1898–2021) to those observed in reference cohort (1978–1986). Dots and shaded bands represent point estimates and associated 95% confidence intervals for incidence rates and relative risks.

Figure 3 presents the APC model findings for the four high-risk endemic areas. In all regions, the risk of dengue incidence gradually increased between the ages of 0 and 9 years, levelled off in subsequent age groups, and then rose significantly in individuals aged 80 years and above. The age effect curves revealed no significant gender-specific variations in the dengue incidence patterns (Figure 3A). Supplementary Figures S5 and S6 detail the age effects across sexes in countries in the four high-risk regions.

Figure 3B illustrates that between 1992 and 2021, period effects indicated an RR of dengue incidence in the three high-risk regions of Southeast Asia, South Asia, and Latin America and the Caribbean. However, the risk of period effects decreased most rapidly in eastern sub-Saharan Africa from 1992 to 2006, while remaining relatively stable in the subsequent decade. In particular, the period risk in Latin America and the Caribbean increased between 1992 and 2014, followed by a subsequent decline. There were also no significant gender differences in the pattern of period effects. Supplementary Figures S7 and S8 detail the period effects across sexes in countries in the four high-risk regions.

Figure 3C demonstrates a global upward trend in the dengue incidence risk across birth cohorts, with particularly pronounced increases observed in successive generational cohorts from South Asia, Southeast Asia, and Latin America and the Caribbean. However, the risk of birth effects declined significantly in eastern sub-Saharan Africa. As with other effects, no marked sex-based variation was observed. Supplementary Figures S9 and S10 detail the cohort effects across sexes in countries in the four high-risk regions.

### 3.5. Age, Period, and Cohort Effects in Exemplary Countries

**Figure 4:**
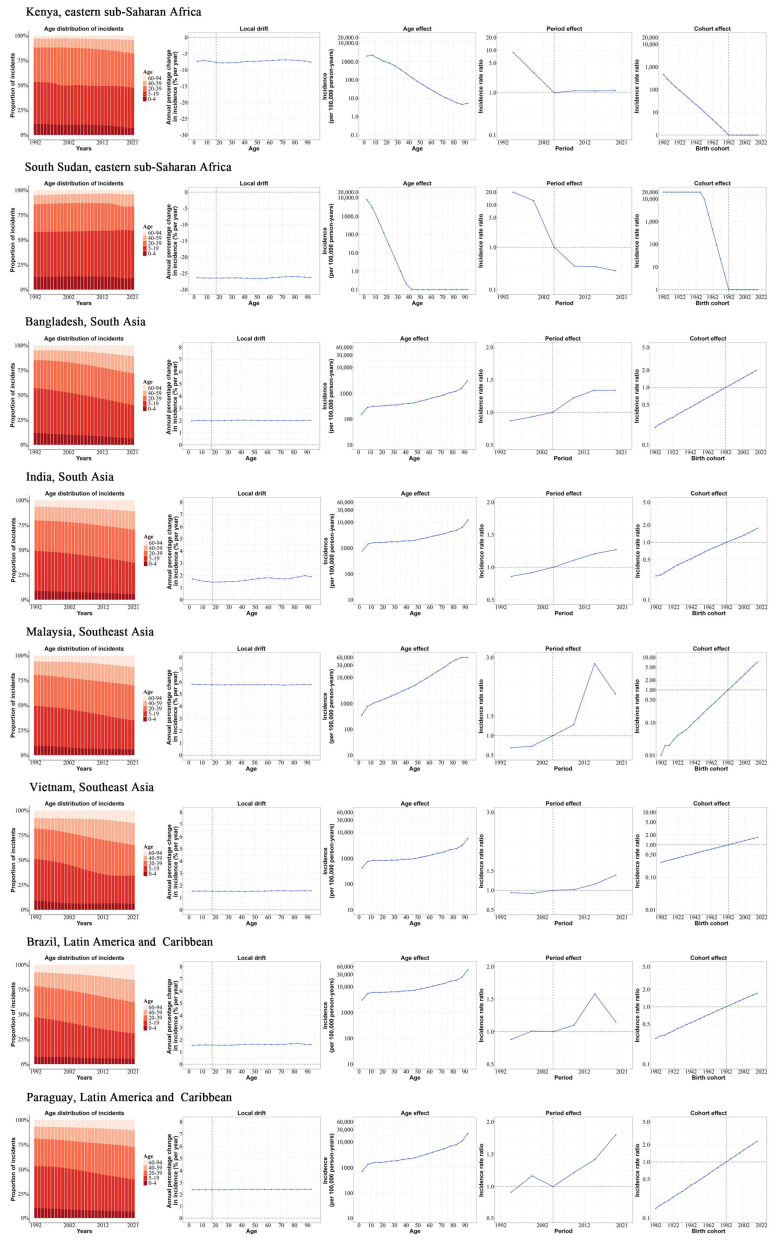
Age–period–cohort effects on exemplary countries across high-risk areas. The age-specific distribution represents the proportion of dengue cases across five broad age groups (0 to 4, 5 to 19, 20 to 39, 40 to 59, and 60 to 94 years) from 1992 to 2021. The local drift represents the estimated annual percentage change in the dengue incidence for each of the 19 age groups (from 0–4 to 90–94 years), derived from age–period–cohort analysis considering the period from 1992 to 2021. Age effects represent age-specific trends in the incidence rates adjusted for temporal variations. Period effects are expressed as the relative risk of incidence, calculated by comparing the age-specific incidence rates during each period to those observed in the reference period (2002–2006). Cohort effects are presented as the relative risks of incidence, calculated by comparing the age-specific incidence rates across birth cohorts (1898–2021) to those observed in the reference cohort (1978–1986). Dots and shaded bands represent point estimates and the associated 95% confidence intervals for the incidence rates and relative risks.

Figure 4 illustrates the age, period, and cohort effects for example countries in high-risk regions. Among these exemplary countries, the majority of cases occurred in individuals under 39 years of age, with a particularly high incidence observed among children, adolescents, and young adults. Among countries with adverse APC effects, Malaysia showed a significant rise in its ASIR, increasing by 187.83% to 2586.69/100,000. It also recorded the highest net drift rate of 5.76% (95% UI: 5.73–5.78), with the risk from APC effects continuing to grow. Brazil exhibited the highest ASIR of 5886.04/100,000, yet it demonstrated a comparatively lower net drift rate of 1.61% (95% UI: 1.59 to 1.62). A similar trend was observed in Bangladesh, India, Vietnam, and Paraguay, with comparable APC effects. However, a contrasting pattern was evident in Malaysia and Brazil, where period effects exhibited a downward trend after 2014. Conversely, countries in eastern sub-Saharan Africa exhibited advantageous APC effects. For instance, in South Sudan, the incidence of dengue fever exhibited a substantial decline, with the net drift measuring −25.79% (95% UI: −26.46 to −25.11), indicating a notable decrease in the risk of APC effects.

## 4. Discussion

Based on data from GBD 2021, this study provided a systematic analysis of the dengue incidence globally and in high-risk regions, offering a comprehensive assessment of the incidence, long-term trends, and regional disparities. The results indicate a sustained rise in the incidence of dengue over the last thirty years. Globally, both the incidence and ASIRs of dengue have demonstrated an increasing trend from 1992 to 2021. A recent study highlighted that countries with the heaviest dengue burden are concentrated in regions with low-to-moderate sociodemographic indices, whereas the burden in developed countries remains relatively low [24], aligning with our findings. This phenomenon reflects differences in public health infrastructure, accessibility, and vector control, significantly influencing the geographical distribution and frequency of dengue cases [4]. Therefore, we focused our analysis on high-risk areas—densely populated, rapidly urbanising regions with limited health resources—where targeted interventions offer a crucial opportunity to mitigate the global disease burden and prioritise health services. This study found that over the past three decades, the dengue incidence has continued to rise in high-risk regions such as Southeast Asia, South Asia, and Latin America and the Caribbean. In contrast, eastern sub-Saharan Africa has shown a marked decline in both the incidence and ASIR during the same period, which aligns with the findings of Deng [25]. We further used an APC analysis to analyse the potential reasons for changes in the dengue incidence risk in different regions.

A noteworthy observation was the slowdown in the rate of increases in the dengue incidence during the COVID-19 pandemic, a trend consistent with monitoring reports from the WHO [26]. This shift may be attributable to several overlapping factors. Public health measures—such as lockdowns, travel restrictions, and social distancing—reduced human mobility and interaction, inadvertently limiting human–mosquito contact and lowering the transmission risk [27]. However, this apparent decline may also reflect disruptions to routine disease surveillance, as health systems were overwhelmed and resources diverted towards COVID-19 responses [28]. A reduced diagnostic capacity and limited access to care likely led to underreporting, contributing to an artificial drop in the recorded dengue cases. Therefore, the trends in the dengue incidence during 2020–2021 should be interpreted with caution.

The APC results revealed that Southeast Asia, South Asia, and Latin America and the Caribbean experienced unfavourable period and cohort effects, resulting in a rising dengue incidence in these high-risk regions. These effects likely reflect the combined influence of multiple factors, including climate change, rapid urbanisation, an increasing population density, and socio-cultural practices. Environmental and climatic changes are important drivers. Global warming enhances mosquito breeding conditions by accelerating the reproductive cycle of *Aedes aegypti*, shortening the virus incubation period within the vector, and expanding its geographical range into previously unsuitable areas, thereby increasing the dengue risk [29,30]. Moreover, climate variability driven by phenomena such as El Niño and La Niña, which result in higher temperatures, increased rainfall, and greater humidity, has been linked to short-term spikes in dengue outbreaks in affected areas [31]. However, researchers such as Paul Reiter have emphasised that historical dengue trends show that climate factors alone rarely dictate transmission. Instead, shifts in human ecology—such as urbanisation, occupations, population migration, urban design, and water management and changes in vector behaviour—have a much more substantial influence than the temperature alone [15,32,33]. This underlines the need to focus on these underlying factors. A rising population density and rapid urbanisation contribute significantly to dengue transmission. In high-risk regions, urban growth has outpaced infrastructure development, leading to increased population mobility and crowding, conditions that facilitate dengue transmission. Over 80% of the Latin American population now resides in urban areas, while urban populations in South and Southeast Asia are growing at rates exceeding 2% annually [34], further heightening the risk of transmission. Socio-cultural factors also play a role. In South and Southeast Asia, practices such as open water storage, shared sanitation systems, and traditional housing provide ideal environments for *Aedes aegypti* to breed [35]. *Aedes aegypti* is a daytime-active mosquito that prefers to bite people indoors or in dark environments. In tropical regions, homes, schools, and workplaces often rely on natural ventilation during the daytime, creating favourable conditions for contact between humans and mosquitoes, thereby facilitating the transmission of dengue [32]. Similarly, in Latin America and the Caribbean, poor water and waste management—often a legacy of unregulated urban expansion—has exacerbated the dengue risk. Human mobility further enhances transmission opportunities, even in the poorest countries. Large-scale road construction and affordable air travel have opened up remote areas for development, increasing the frequency of inter-regional travel [36]. It is worth noting that the increase in the incidence may also reflect improved disease surveillance. Over the past three decades, many countries have enhanced their reporting systems through the use of digital health records, better laboratory capacities, and public education. India’s Integrated Disease Surveillance Programme and real-time alert platforms in Southeast Asia using mobile apps and GIS have strengthened case detection and data accuracy [37]. As a result, some of the observed rise in the incidence may have been due to better reporting rather than a true epidemiological increase.

Notably, eastern sub-Saharan Africa exhibited favourable period and cohort effects, indicating a declining risk of dengue incidence over the past 30 years. The reasons for this decline are likely multifactorial. Studies have suggested that in parts of eastern sub-Saharan Africa, reduced rainfall and changes in seasonal precipitation patterns may have constrained mosquito breeding, thereby limiting viral transmission [38]. South Sudan, often considered representative of this region, has demonstrated the most pronounced decline in the dengue incidence [39]. This trend may also be attributed to the region’s predominantly highland and hilly terrain, combined with prolonged droughts and extreme weather conditions, which have likely inhibited the proliferation of *Aedes aegypti* [40]. Moreover, the pattern of dispersed settlement expansion in sub-Saharan Africa has resulted in built-up areas growing faster than the urban population, leading to a declining population density in urbanised zones [41]. This relative sparsity may help limit the speed and intensity of dengue transmission. Importantly, sub-Saharan Africa is the region most affected by malaria. Due to overlapping clinical symptoms and a diagnostic paradigm heavily focused on malaria, dengue is frequently misdiagnosed or overlooked [42,43]. Routine dengue diagnostics are not formally integrated into many national health systems, and surveillance is largely limited to outbreak investigations rather than continuous monitoring [44]. Thus, while the data suggest a decline in the dengue incidence, underdiagnosis and underreporting may have led to an overestimation of this downward trend.

Our study shows that the net drift rate of dengue in Brazil is relatively low among countries with unfavourable APC effects, but Brazil is currently one of the most affected countries. A related Brazilian study has also demonstrated the relationship between high temperatures and dengue, showing that increased rates of hospitalisation for dengue were primarily driven by elevated temperatures. This association is linked to the mosquito life cycle and increased vector activity under warmer conditions [45]. In the Lao People’s Democratic Republic, where dengue was first reported in 1979, the cases and incidence have risen significantly, with densely populated areas showing the highest rates [46]. In the present study, Malaysia was identified as the nation with the highest net dengue drift rate and the most rapid escalation in its APC effect. This finding is consistent with the observations reported by Yusoff et al., who noted a marked rise in the dengue incidence in Malaysia, particularly in Penang [47]. Factors associated with an increased dengue incidence include higher *Aedes* indices, the presence of Priority Area 1 zones, and the number of first visits to government health clinics. These results highlight the importance of proactive vector management and early preventive measures in high-risk areas to mitigate the outbreak risk, and focusing on the high risk of incidence in example countries can provide a direction for dengue control.

Our study found that, globally and within high-risk regions, children and adolescents aged 5–19 years and young adults under 39 years of age were the primary groups affected by dengue. This phenomenon may be attributed to several factors, including a high percentage of young people, rapid urban population growth, large-scale population movements, and inadequate financial resources [48]. Moreover, the immature immune systems of younger individuals may render them more susceptible to dengue infection. This highlights the need to further strengthen vaccination efforts [48]. Another possible reason for the higher incidence in the adult population is the dynamics of immunity levels within the population. After a large outbreak, the transmission levels typically decrease in the following years, leading to a decline in immunity levels among adults. This results in a shift in infection to slightly older age groups [49,50]. Although the proportion of cases in older age groups remains relatively small, the age effect suggests that they are at the highest risk of developing the disease. For instance, studies have demonstrated a rise in the mean age of dengue patients in countries such as Bangladesh and India [51,52], a finding that aligns with the observations made in the selected countries. Our study identified both children and older adults—especially those aged over 80—as high-risk groups, with the latter showing a notably elevated susceptibility to dengue. The susceptibility of the elderly to dengue may involve the following factors: First, with age, physiological functions gradually decline, and the immune system’s capacity becomes progressively suppressed [53]. Secondly, older people are more likely to contract secondary dengue. A study conducted in Singapore found that adults aged 60 and above experienced nearly double the rate of secondary dengue infections compared to younger cohorts [54]. Third, the widespread occurrence of chronic diseases in older populations may elevate their vulnerability; a meta-analysis revealed a significantly heightened risk of severe dengue among individuals with diabetes [55]. The WHO has sought to address this trend by promoting integrated vector management strategies in the Americas and Southeast Asia. However, the rising age of dengue onset is expected to persist [56]. In summary, as dengue’s epidemiological characteristics have evolved, the elderly have emerged as a high-risk group. Future efforts should focus on developing targeted prevention and management approaches to alleviate the disease burden and enhance outcomes in these vulnerable groups.

This study has several limitations. Firstly, the GBD 2021 dengue estimates were derived from secondary sources, and in the absence of timely or robust case reports, the incidence estimates were derived using the available information and modelling techniques. Therefore, some of the estimates may have had wide confidence intervals (CIs), particularly for earlier time periods and age strata with limited case numbers. Secondly, the segmented treatment of the data inevitably resulted in some data loss; grouping data into five-year intervals for APC modelling may have obscured subtle trends in the age, period, or cohort dimensions. To minimise data loss, we used a five-year moving average for the calculations, striking a balance between data completeness and analytical precision. Despite these limitations, the present study offers valuable insights into the epidemiological characterisation of dengue and provides directions for future in-depth research.

## 5. Conclusions

The global incidence of dengue has been steadily rising over the past three decades, positioning it as a pressing international public health concern. Among high-risk regions, only eastern sub-Saharan Africa has experienced a decline. While children and younger adults are primarily affected, the risk to older populations should not be overlooked. The unfavourable period and cohort effects suggest that the dengue incidence is likely to continue rising in most high-risk regions. The limited effectiveness of existing dengue prevention and management strategies in most high-risk areas highlights the critical need for more focused and effective interventions to combat this escalating health threat.

## Figures and Tables

**Table 1:** Trends in dengue incidence across high-risk areas, 1992–2021.

	Global		Eastern Sub-Saharan Africa		South Asia		Southeast Asia		Latin America and Caribbean	
	1992	2021	1992	2021	1992	2021	1992	2021	1992	2021
Population										
Number, n × 1,000,000	5497 (5379 to 5624)	7891 (7667 to 8131)	202 (196 to 208)	426 (406 to 447)	1136 (1069 to 1205)	1847 (1668 to 2039)	482 (463 to 501)	698 (670 to 728)	405 (391 to 419)	594 (560 to 626)
Percentage of global population, %	100.0	100.0	3.67	5.40	20.67	23.41	8.1	8.85	7.37	7.53
Incident cases										
Number, n × 100,000	288.47 (62.07 to 530.97)	589.64 (154.73 to 1068.85)	23.07 (1.76 to 57.35)	3.88 (0.07 to 14.62)	134.08 (2.08 to 278.03)	318.12 (18.72 to 670.71)	28.64 (7.35 to 62.97)	67.28 (47.88 to 104.31)	90.6 (12.17 to 196.14)	165.49 (65.78 to 305.44)
Percentage of global cases, %	100.0	100.0	8.0	0.7	46.48	53.95	9.93	11.41	31.41	28.07
Percent change for 1992–2021, %	104.40		−83.20		137.26		134.90		82.65	
Percent change for 2019–2021, %	3.81		3.88		3.22		6.46		3.04	
All-age incidence rate										
Rate per 100,000	524.76 (112.91 to 965.89)	747.2 (196.08 to 1354.46)	1143.51 (87.08 to 2842.94)	90.97 (1.54 to 343.2)	1180.36 (18.33 to 2447.58)	1722.77 (101.4 to 3632.17)	594.63 (152.64 to 1307.19)	963.54 (685.65 to 1493.81)	2236.28 (300.3 to 4841.16)	2785.41 (1107.14 to 5141.04)
Percent change for 1992–2021, %	42.39		−92.04		45.95		62.04		24.56	
Percent change for 2019–2021, %	1.89		−0.80		1.03		4.39		1.53	
ASIRs										
Rate per 100,000	510.67 (108.43 to 941.74)	752.04 (196.33 to 1363.35)	1142.19 (87.44 to 2843.11)	94.37 (1.55 to 364.39)	1182.78 (18.6 to 2452.17)	1726.94 (102.48 to 3635.94)	587.51 (150.84 to 1281.39)	971.89 (691.33 to 1500.41)	2235.8 (298.96 to 4852.39)	2781.19 (1107.73 to 5129.38)
Percent change for 1992–2021, %	47.26		−91.74		46.01		65.43		24.39	
Percent change for 2019–2021, %	1.85		−0.33		1.05		4.41		1.46	
APC model estimates										
Net drift of ASIR for 1992–2021, % per year	2.14 (2.1 to 2.19)		−9.02 (−9.07 to −8.98)		1.57 (1.56 to 1.58)		2.47 (2.44 to 2.51)		1.78 (1.76 to 1.8)	

Notes: The all-age incidence rate refers to the crude (unadjusted) incidence. The age-standardised incidence rate is calculated using the direct standardisation method, applying the global standard population defined in the GBD 2021 study. The net drift represents the estimated average annual percentage change in the ASIR over time, as modelled by the age–period–cohort analysis. The values in parentheses for all GBD-related health indicators denote 95% uncertainty intervals, while those reported for the net drift refer specifically to 95% confidence intervals. APC: age–period–cohort. ASIRs: age-standardised incidence rates.

## Data Availability

The datasets supporting this study are freely available at https://vizhub.healthdata.org/gbd-results/ (accessed on 15 January 2025).

## Supplementary Materials

The following supporting information can be downloaded at https://www.mdpi.com/article/10.3390/tropicalmed10060173/s1: Figure S1: The local drifts of the dengue incidence rate for both sexes in countries in eastern sub-Saharan Africa, South Asia, and Southeast Asia, 1992–2021; Figure S2: The local drifts of the dengue incidence rate for both sexes in Latin American and Caribbean countries, 1992–2021; Figure S3: Age distribution of dengue incidence for both sexes in countries in eastern sub-Saharan Africa, South Asia, and Southeast Asia, 1992–2021; Figure S4: Age distribution of dengue incidence for both sexes in Latin American and Caribbean countries, 1992–2021; Figure S5: Age effects on dengue incidence rate for both sexes in countries in eastern sub-Saharan Africa, South Asia, and Southeast Asia; Figure S6: Age effects on dengue incidence rate for both sexes in Latin American and Caribbean countries; Figure S7: Period effects on dengue incidence rate for both sexes in countries in eastern sub-Saharan Africa, South Asia, and Southeast Asia; Figure S8: Period effects on dengue incidence rate for both sexes in Latin American and Caribbean countries; Figure S9: Cohort effects on dengue incidence rate for both sexes in countries in eastern sub-Saharan Africa, South Asia, and Southeast Asia; Figure S10: Cohort effects on dengue incidence rate for both sexes in Latin American and Caribbean countries; Table S1: List of countries in high-risk regions; Table S2: The Lexis diagram of the dengue data for the APC model; Table S3: Time trends in dengue incidence rate for both sexes in countries in high-risk areas, 1992–2021.

## Abbreviations

The following abbreviations are used in this manuscript:

APC	Age–period–cohort
ASIRs	Age-standardised incidence rates
CI	Confidence interval
DALY	Disability-adjusted life year
GBD	Global Burden of Disease Study
ICD	International Classification of Diseases
RR	Relative risk
UI	Uncertainty interval
WHO	World Health Organization
