# Supplementary material for: Epidemiological Trends and Age–Period–Cohort Effects on Dengue Incidence Across High-Risk Regions from 1992 to 2021
Source: Trop Med Infect Dis. 2025 Jun 18;10(6):173. doi: 10.3390/tropicalmed10060173 (PMC12197733; doi:10.3390/tropicalmed10060173)
Supplement: Supplementary file 1 [file tropicalmed-10-00173-s001.zip › tropicalmed-3604039-supplementary.pdf]

Supplementary materials

Figure S1 The local drifts of dengue incidence rate for both sexes in Eastern Sub-Saharan Africa, South Asia and Southeast Asia countries, 1992-2021 1

Figure S2 The local drifts of dengue incidence rate for both sexes in Latin America and Caribbean countries, 1992-2021 2

Figure S3 Age distribution of incidences for both sexes from dengue in Eastern Sub-Saharan Africa, South Asia and Southeast Asia countries, 1992-2021 3

Figure S4 Age distribution of incidences for both sexes from dengue in Latin America and Caribbean countries, 1992-2021 4

Figure S5 Age effects on dengue incidence rate for both sexes in Eastern Sub-Saharan Africa, South Asia and Southeast Asia countries 5

Figure S6 Age effects on dengue incidence rate for both sexes in Latin America and Caribbean countries 6

Figure S7 Period effects on dengue incidence rate for both sexes in Eastern Sub-Saharan Africa, South Asia and Southeast Asia countries 7

Figure S8 Period effects on dengue incidence rate for both sexes in Latin America and Caribbean countries 8

Figure S9 Cohort effects on dengue incidence rate for both sexes in Eastern Sub-Saharan Africa, South Asia and Southeast Asia countries 9

Figure S10 Cohort effects on dengue incidence rate for both sexes in Latin America and Caribbean countries 10

Table S1 List of country compositions in high-risk regions 13

Table S2 The lexis diagram of dengue data for the APC model 13

Table S3 Time trends in dengue incidence rate for both sexes in high-risk area countries, 1992-2021 13

Figure S1 The local drifts of dengue incidence rate for both sexes in Eastern Sub-Saharan Africa, South Asia and Southeast Asia countries, 1992-2021

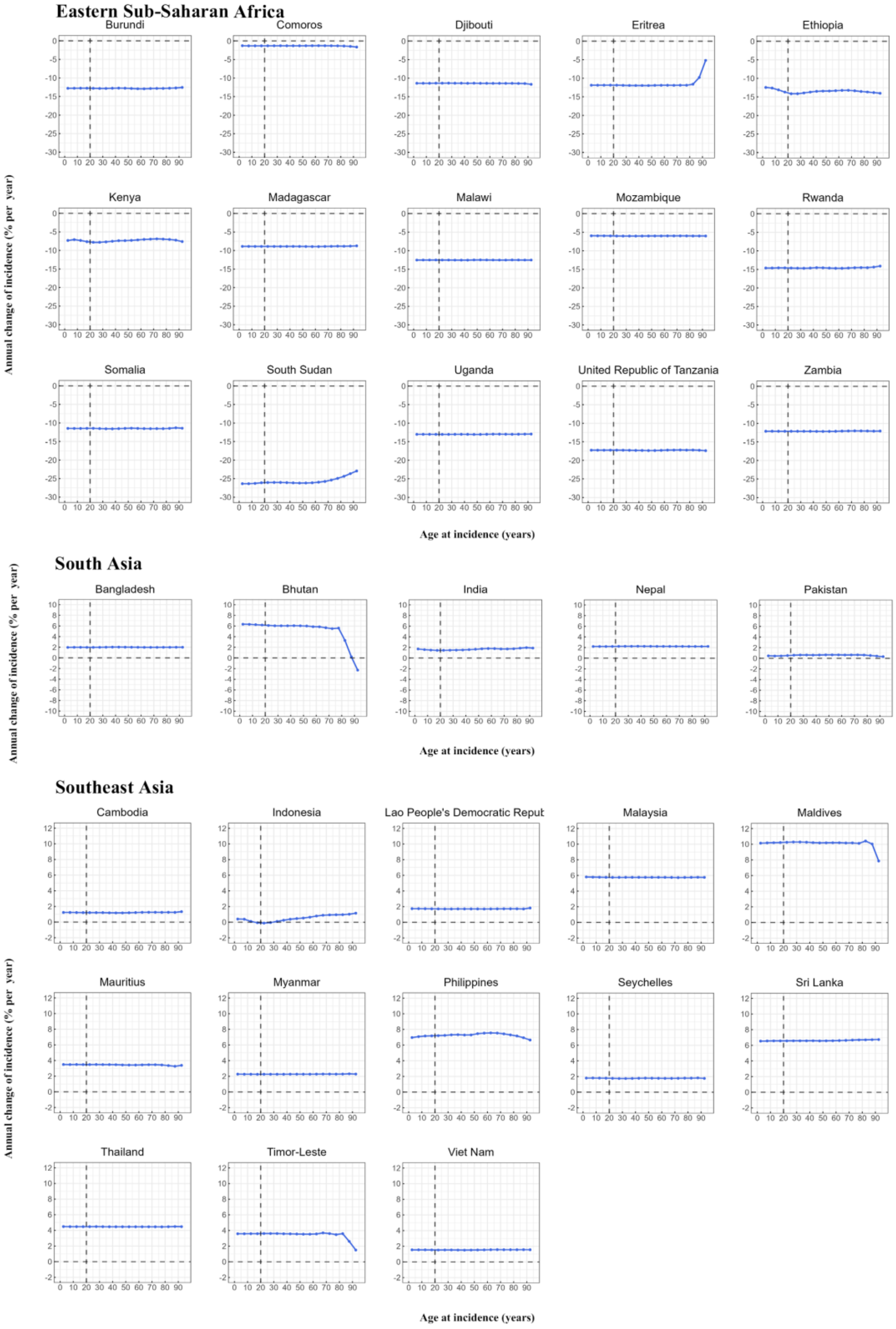

Figure S2 The local drifts of dengue incidence rate for both sexes in Latin America and Caribbean countries, 1992-2021

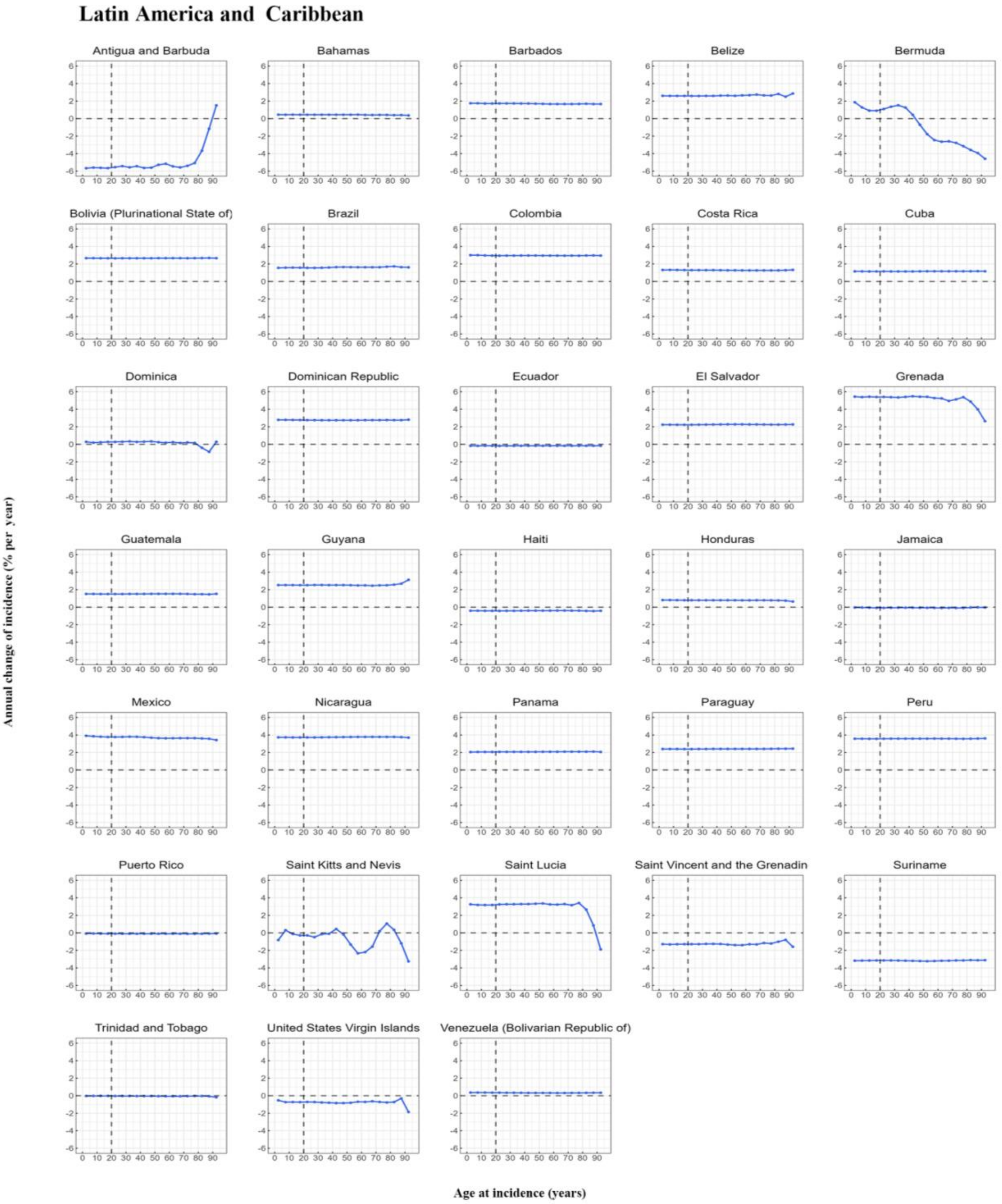

Figure S3 Age distribution of incidences for both sexes from dengue in Eastern Sub-Saharan Africa, South Asia and Southeast Asia countries, 1992-2021

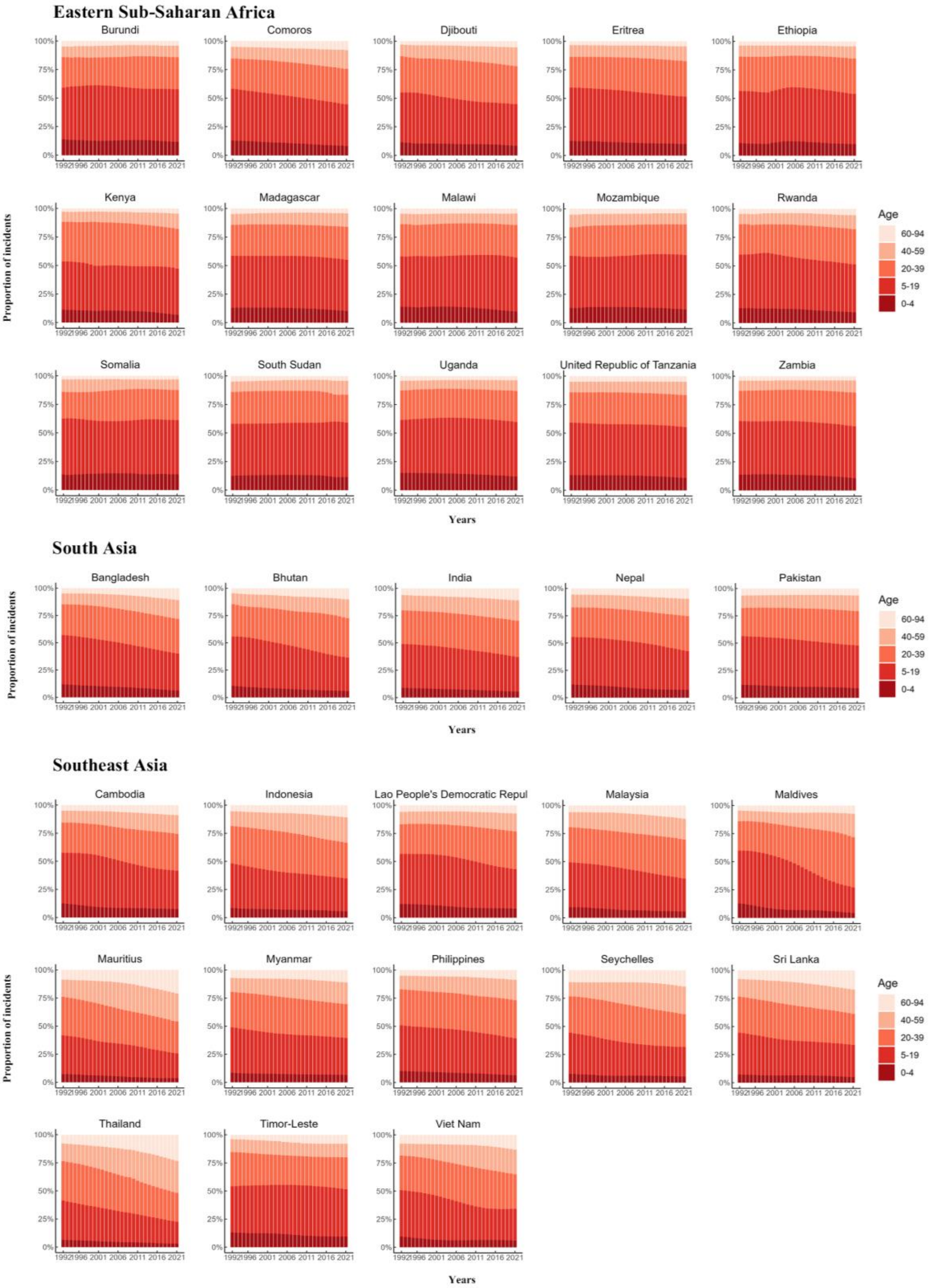

Figure S4 Age distribution of incidences for both sexes from dengue in Latin America and Caribbean countries, 1992-2021

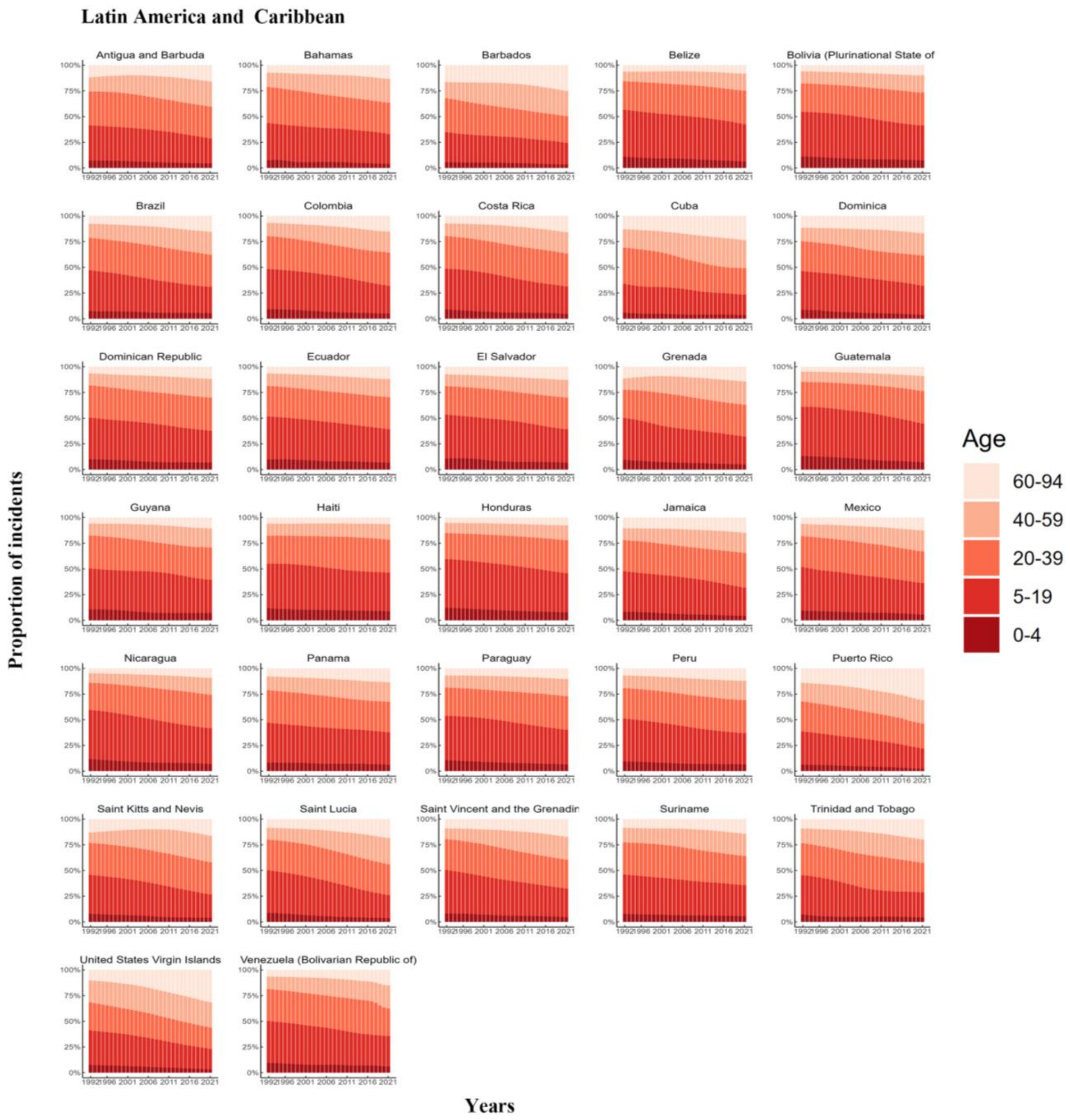

Figure S5 Age effects on dengue incidence rate for both sexes in Eastern Sub-Saharan Africa, South Asia and Southeast Asia countries

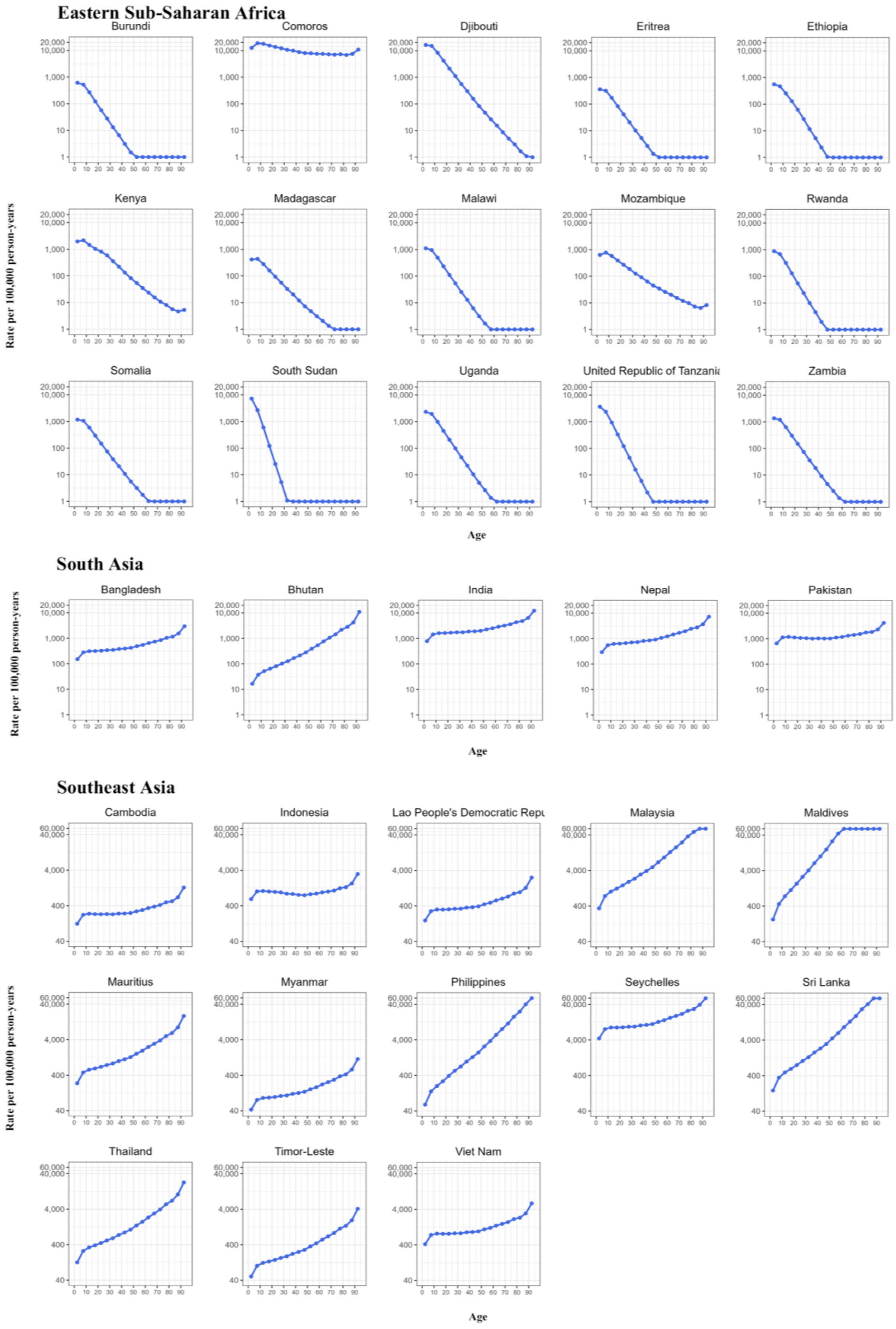

Figure S6 Age effects on dengue incidence rate for both sexes in Latin America and Caribbean countries

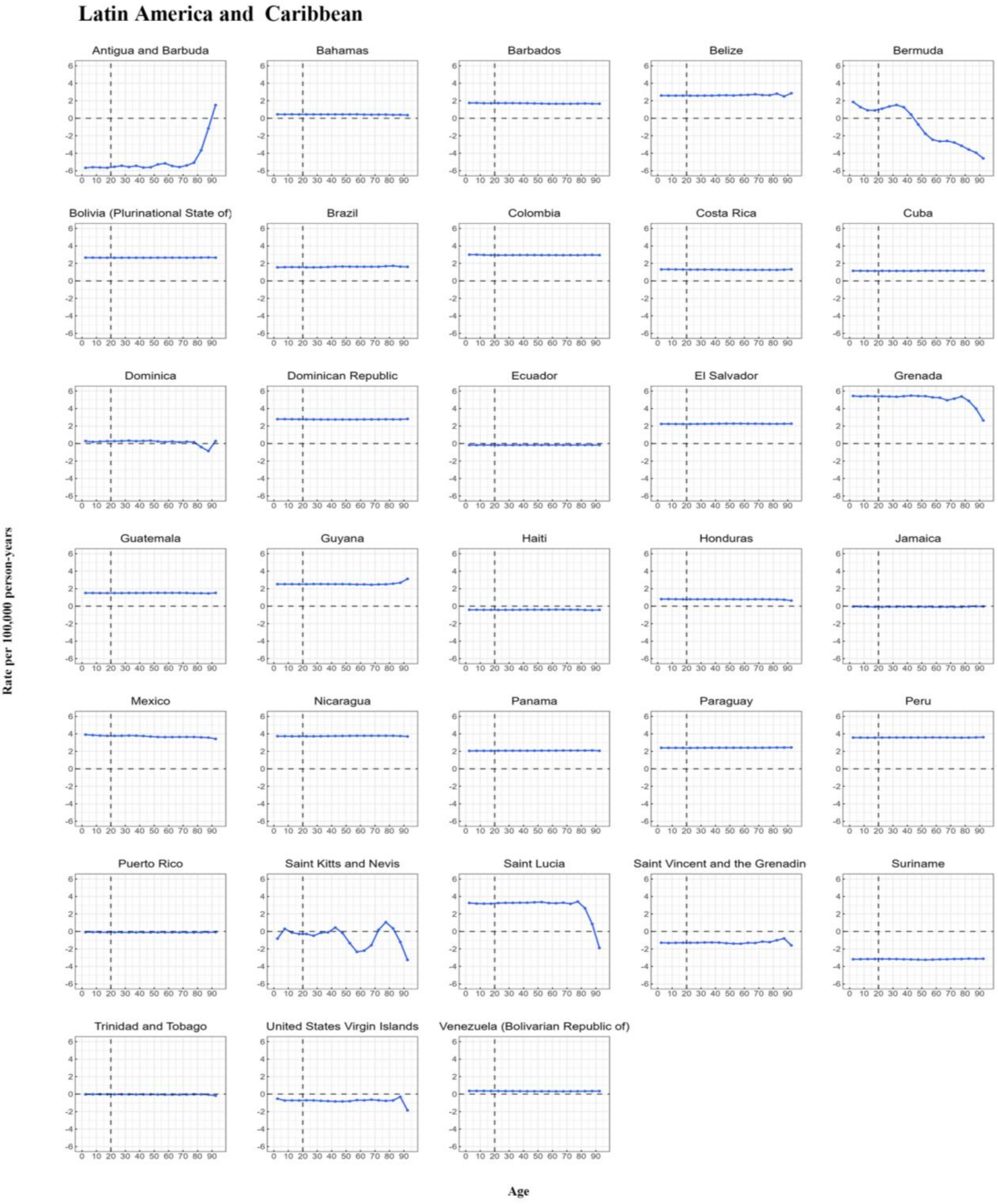

Figure S7 Period effects on dengue incidence rate for both sexes in Eastern Sub-Saharan Africa, South Asia and Southeast Asia countries

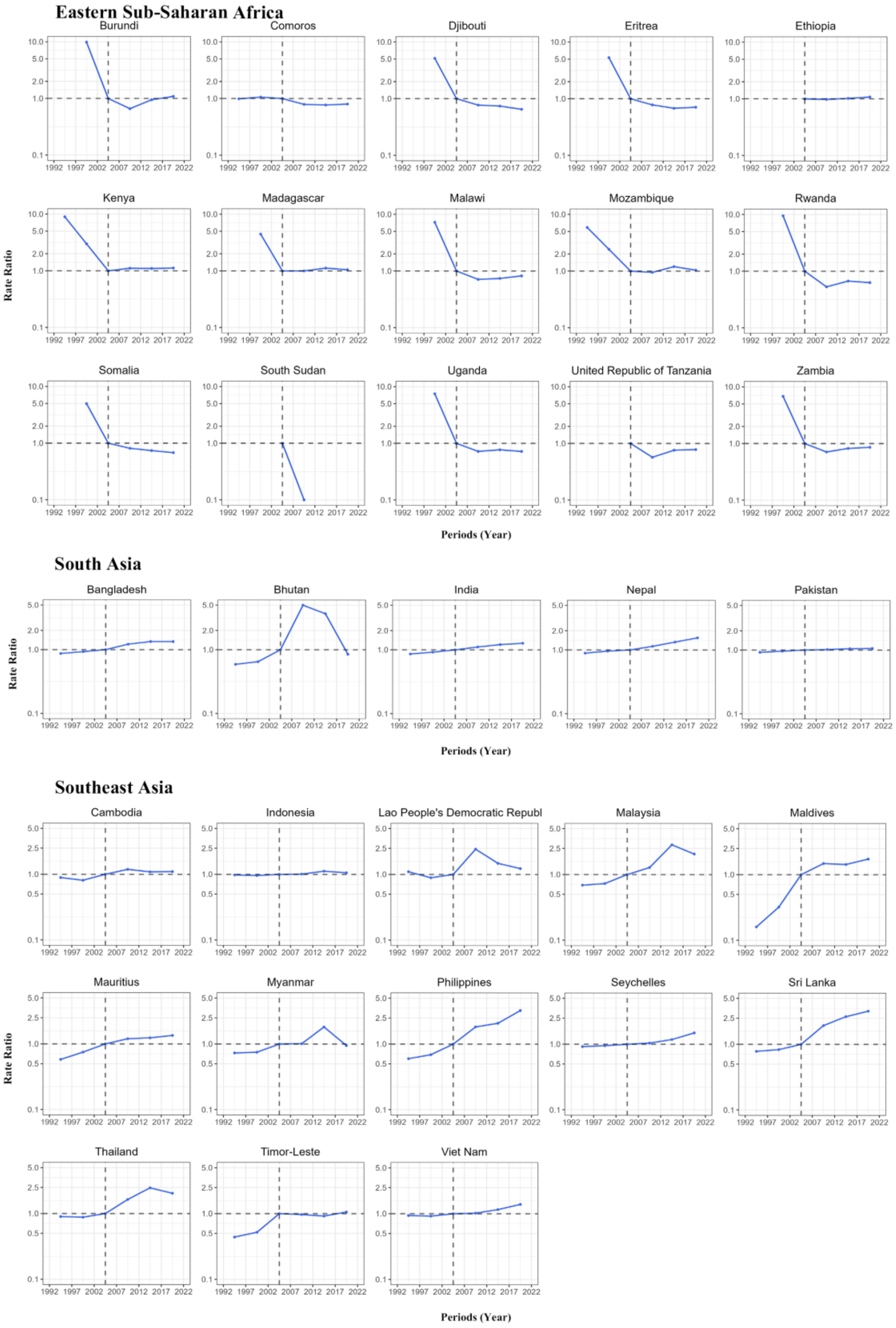

Figure S8 Period effects on dengue incidence rate for both sexes in Latin America and Caribbean countries

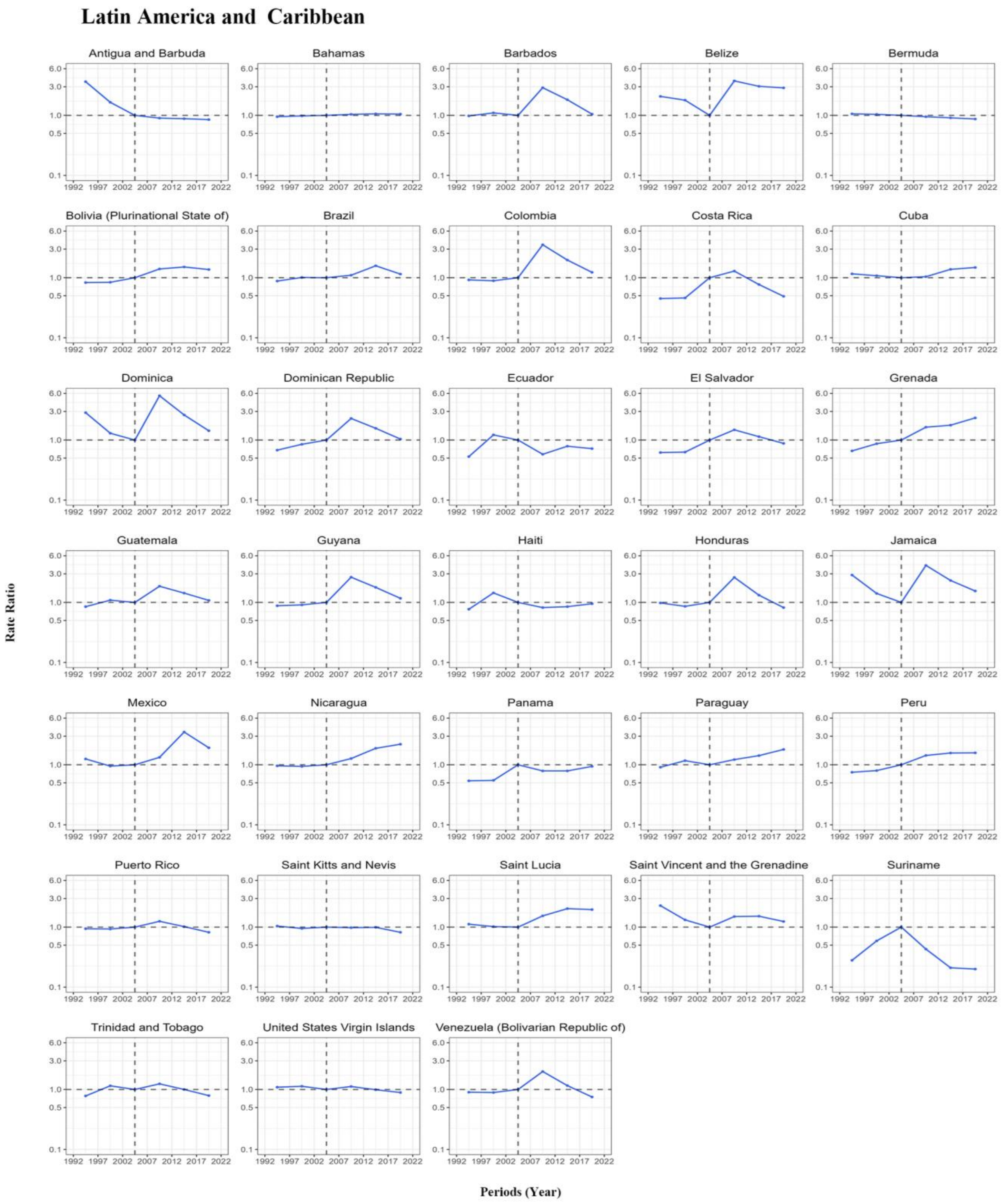

Figure S9 Cohort effects on dengue incidence rate for both sexes in Eastern Sub-Saharan Africa, South Asia and Southeast Asia countries

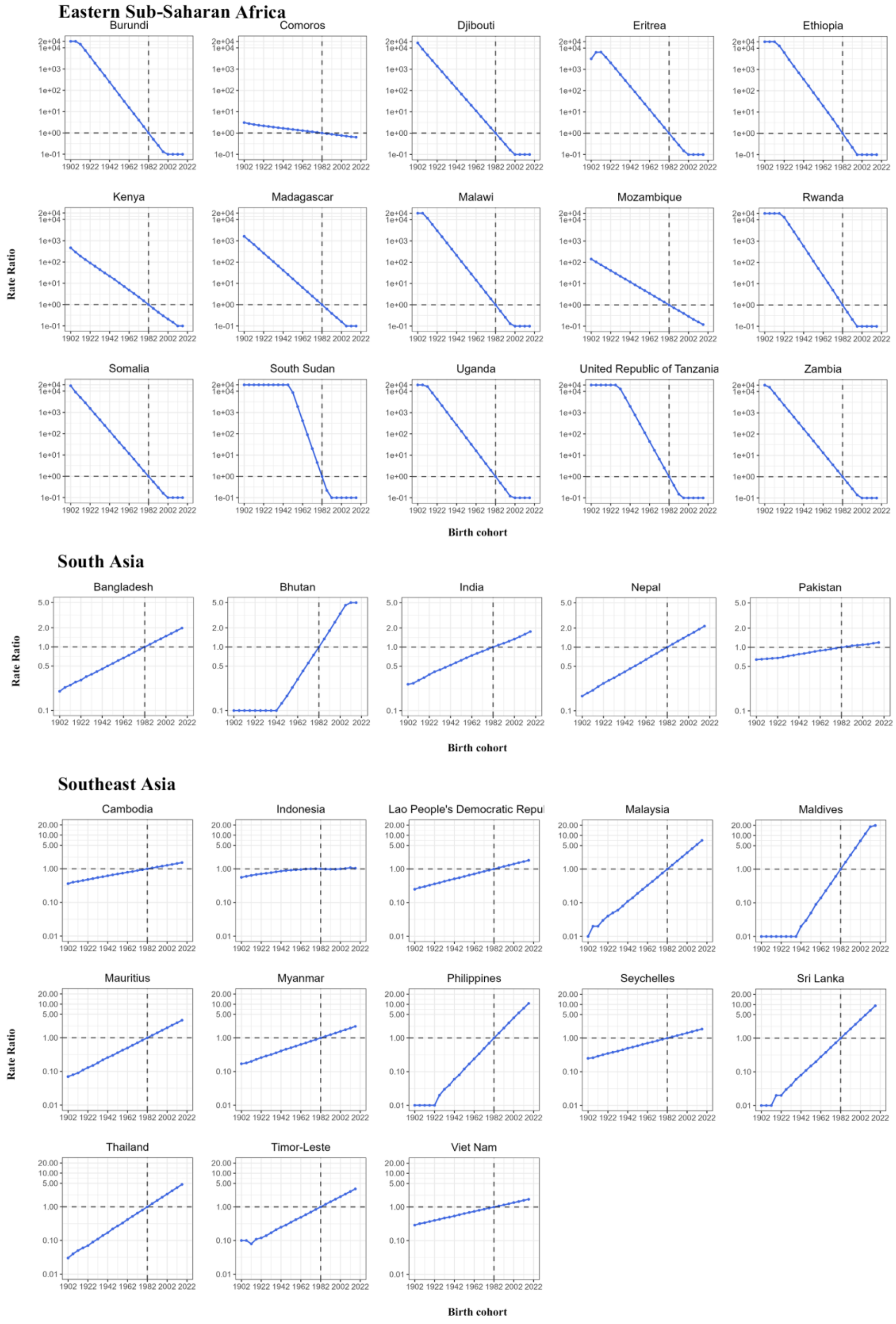

Figure S10 Cohort effects on dengue incidence rate for both sexes in Latin America and Caribbean countries

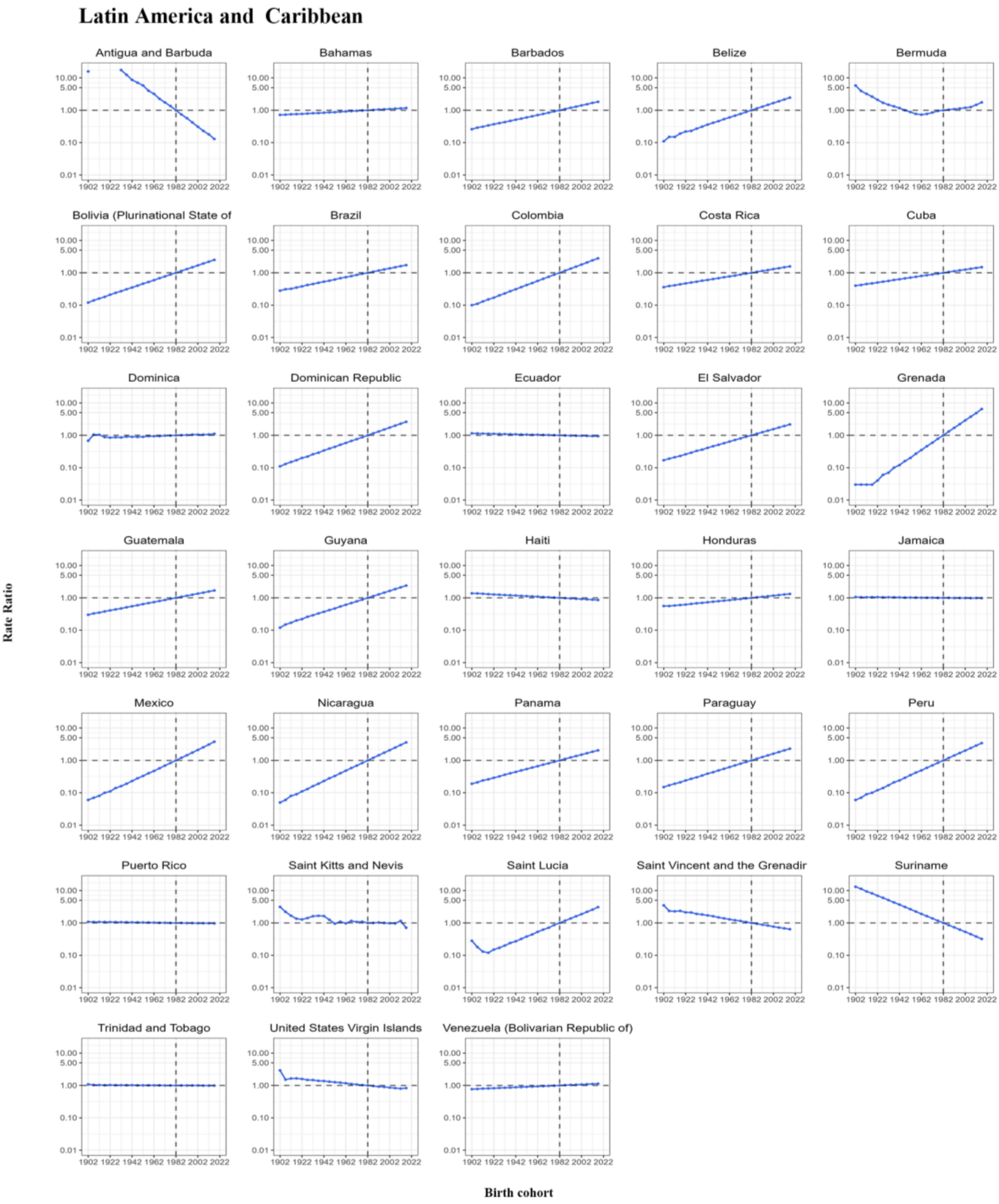

Table S1 List of country compositions in high-risk regions

**Regions**	**Countries**
Eastern Sub-Saharan Africa (15)	Burundi, Comoros, Djibouti, Eritrea, Ethiopia, Kenya, Madagascar, Malawi, Mozambique, Rwanda, Somalia, South Sudan, Uganda, United Republic of Tanzania, Zambia
South Asia (5)	Bangladesh, Bhutan, India, Nepal, Pakistan
Southeast Asia (13)	Cambodia, Indonesia, Lao People's Democratic Republic, Malaysia, Maldives, Mauritius, Myanmar, Philippines, Seychelles, Sri Lanka, Thailand, Timor-Leste, Viet Nam
Latin America and Caribbean (33)	Antigua and Barbuda, Bahamas, Barbados, Belize, Bermuda, Bolivia (Plurinational State of), Brazil, Colombia, Costa Rica, Cuba, Dominica Dominican Republic, Ecuador, El Salvador, Grenada, Guatemala, Guyana, Haiti, Honduras, Jamaica, Mexico, Nicaragua, Panama, Paraguay, Peru, Puerto Rico, Saint Kitts and Nevis, Saint Lucia, Saint Vincent and the Grenadines, Suriname, Trinidad and Tobago, United States, Virgin Islands, Venezuela (Bolivarian Republic of)

Table S2 The lexis diagram of dengue data for the APC model

	**Age groups**																			
**Period (five-year average)**	0-4	5-9	10-14	15-19	20-24	25-29	30-34	35-39	40-44	45-49	50-54	55-59	60-64	65-69	70-74	75-79	80-84	85-89	90-94	Birth cohort
																			X	1898-1906
																		X	X	1903-1911
																	X	X	X	1908-1916
																X	X	X	X	1913-1921
															X	X	X	X	X	1918-1926
														X	X	X	X	X	X	1923-1931
													X	X	X	X	X	X		1928-1936
												X	X	X	X	X	X			1933-1941
											X	X	X	X	X	X				1938-1946
										X	X	X	X	X	X					1943-1951
									X	X	X	X	X	X						1948-1956
								X	X	X	X	X	X							1953-1961
							X	X	X	X	X	X								1958-1966
						X	X	X	X	X	X									1963-1971
					X	X	X	X	X	X										1968-1976
				X	X	X	X	X	X											1973-1981
			X	X	X	X	X	X												1978-1986
		X	X	X	X	X	X													1983-1991
1992-1996	**X**[a]	X	X	X	X	X														1988-1996
1997-2001	X	X	X	X	X															1993-2001
2002-2006	X	X	X	X																1998-2006
2007-2011	X	X	X																	2003-2011
2012 -2016	X	X																		2008-2016
2017- 2021	X																			2013-2021

Note: X denotes incidence rate data of each age group from the corresponding period.

[a]For instance, the incidence rate of age 0-4 years in 1992-1996 interval is filled in the square with a bold X (see table), and this square belongs to the cohort 1988-1996.

Table S3 Time trends in dengue incidence rate for both sexes in high-risk area countries, 1992-2021

Region		Population		Incident		All age incidence		Age standardized incidence		
**Area**	**Country**	**Number in 2021**	**Percent change 1992-2021, %**	**Number in 2021**	**Percent change 1992-2021, %**	**Rate in 2021 (per 100,000)**	**Percent change 1992-2021, %**	**Rate in 2021 (per 100,000)**	**Percent change 1992-2021, %**	**Net drift of incidence rate, % per year**
**Eastern sub-Saharan Africa**										
	Burundi	13220294	127.38	2304 (39 to 15878)	-91.5	17.43 (0.29 to 120.1)	-96.26	17.27 (0.29 to 119)	-96.29	-12.8 (-13.05 to -12.55)
	Comoros	744374	54.3	88298 (17 to 589727)	40.55	11862.03 (2.31 to 79224.53)	-8.91	11841.48 (2.3 to 78807.32)	-8.43	-1.3 (-1.36 to -1.24)
	Djibouti	1258687	155.97	7677 (66 to 54013)	-86.4	609.9 (5.24 to 4291.21)	-94.69	614.93 (5.29 to 4326.66)	-94.61	-11.38 (-11.52 to -11.24)
	Eritrea	6597908	109	690 (3 to 5014)	-91.04	10.45 (0.05 to 76)	-95.71	10.46 (0.05 to 76.04)	-95.71	-11.7 (-12.13 to -11.27)
	Ethiopia	108938000	98.5	16462 (172 to 79767)	-94.04	15.11 (0.16 to 73.22)	-97	14.9 (0.17 to 71.88)	-97.07	-13.52 (-13.67 to -13.37)
	Kenya	50062396	103.3	169905 (2884 to 1009758)	-73.28	339.39 (5.76 to 2017)	-86.86	333.98 (5.54 to 1966.9)	-87.04	-7.32 (-7.41 to -7.22)
	Madagascar	28557425	127.34	9764 (34 to 73349)	-80.37	34.19 (0.12 to 256.85)	-91.37	33.87 (0.12 to 254.41)	-91.4	-8.88 (-9 to -8.75)
	Malawi	19447841	85.48	6115 (64 to 41670)	-92.3	31.44 (0.33 to 214.27)	-95.85	30.91 (0.33 to 210.64)	-95.92	-12.52 (-12.67 to -12.38)
	Mozambique	31072654	129.04	35949 (63 to 264455)	-65.04	115.69 (0.2 to 851.08)	-84.74	114.33 (0.2 to 841.09)	-84.89	-6 (-6.07 to -5.93)
	Rwanda	13270033	76.23	1558 (69 to 10399)	-95.92	11.74 (0.52 to 78.37)	-97.68	11.69 (0.52 to 78.01)	-97.68	-14.59 (-14.84 to -14.34)
	Somalia	21606521	178.85	8760 (26 to 67738)	-86.63	40.54 (0.12 to 313.51)	-95.21	40.42 (0.12 to 312.55)	-95.2	-11.49 (-11.65 to -11.33)
	South Sudan	9672522	58.19	93 (4 to 526)	-99.79	0.96 (0.04 to 5.44)	-99.87	0.95 (0.04 to 5.39)	-99.87	-25.79 (-26.46 to -25.11)
	Uganda	43315053	135.11	21738 (266 to 178218)	-92.09	50.19 (0.61 to 411.45)	-96.64	49.58 (0.6 to 406.47)	-96.67	-13 (-13.08 to -12.92)
	United Republic of Tanzania	58447852	113.34	9740 (225 to 82108)	-98.05	16.67 (0.38 to 140.48)	-99.09	16.52 (0.38 to 139.28)	-99.09	-17.28 (-17.36 to -17.19)
	Zambia	19515271	134.89	8239 (124 to 53053)	-90.11	42.22 (0.63 to 271.85)	-95.79	41.76 (0.63 to 268.92)	-95.8	-12.11 (-12.24 to -11.98)

**South Asia**										
	Bangladesh	164636116	44.84	700104 (115272 to 1361863)	144.05	425.24 (70.02 to 827.2)	68.49	425.31 (70.01 to 827.39)	70.23	2 (1.98 to 2.03)
	Bhutan	756900	17.57	392 (10 to 1105)	86.76	51.8 (1.37 to 146.01)	58.86	52.04 (1.38 to 146.69)	61.63	5.55 (4.69 to 6.41)
	India	1414494975	59.78	28205519 (1415602 to 60205863)	137.08	1994.03 (100.08 to 4256.35)	48.38	1997.18 (100.91 to 4261.45)	48.94	1.65 (1.64 to 1.66)
	Nepal	31129812	53.41	276880 (2739 to 813634)	162.42	889.44 (8.8 to 2613.68)	71.06	887.98 (8.79 to 2609.38)	71.22	2.23 (2.19 to 2.27)
	Pakistan	235553608	103	2629294 (53357 to 7216114)	135.09	1116.22 (22.65 to 3063.47)	15.81	1125.5 (22.41 to 3092.4)	16.78	0.59 (0.58 to 0.61)
**Southeast Asia**										
	Cambodia	17044077	57.49	42193 (8733 to 92202)	96.49	247.55 (51.24 to 540.96)	24.77	249.2 (51.84 to 544.45)	26	1.22 (1.12 to 1.32)
	Indonesia	278914182	46.44	2488470 (976958 to 5092593)	43.82	892.2 (350.27 to 1825.86)	-1.79	906.32 (356.12 to 1850.51)	1.77	0.47 (0.42 to 0.53)
	Lao People's Democratic Republic	7377552	68	23740 (5984 to 48972)	122.73	321.79 (81.11 to 663.8)	32.58	323.91 (81.57 to 669.51)	33.9	1.72 (1.58 to 1.86)
	Malaysia	31812226	69.48	816195 (308260 to 2067784)	382.09	2565.67 (969 to 6499.97)	184.45	2586.69 (976.79 to 6552.92)	187.83	5.76 (5.73 to 5.78)
	Maldives	517208	119.04	25895 (0 to 93392)	2871	5006.76 (0 to 18057.04)	1256.37	5095.16 (0 to 18143.76)	1292.41	10.16 (9.81 to 10.51)
	Mauritius	1271964	13.42	12713 (347 to 51033)	215.61	999.46 (27.29 to 4012.12)	178.26	1009.32 (27.58 to 4044.79)	179.67	3.46 (3.32 to 3.61)
	Myanmar	56419670	36.07	44433 (9641 to 98009)	43.13	78.76 (17.09 to 173.71)	5.18	79.26 (17.15 to 174.81)	6.64	2.28 (2.21 to 2.36)
	Philippines	113249536	71.18	1262723 (299748 to 4486387)	973	1114.99 (264.68 to 3961.51)	526.81	1111.2 (265.17 to 3936.42)	529.71	7.32 (7.29 to 7.36)
	Seychelles	105431	41.67	12844 (2396 to 54246)	127.61	12182.01 (2272.69 to 51451.16)	60.67	12417.92 (2317.99 to 52444.37)	65.47	1.78 (1.64 to 1.92)
	Sri Lanka	22270055	27.43	466306 (122172 to 1557129)	472.76	2093.87 (548.59 to 6992.03)	349.47	2106.45 (552.08 to 7025.59)	353.19	6.61 (6.57 to 6.64)
	Thailand	66683246	14.33	416690 (114971 to 918118)	116.64	624.88 (172.41 to 1376.83)	89.48	630.4 (174.78 to 1381.13)	91.39	4.48 (4.45 to 4.5)
	Timor-Leste	1397733	71.64	3125 (826 to 8007)	360.19	223.56 (59.08 to 572.84)	168.11	221.18 (58.52 to 566.7)	163.9	3.51 (2.99 to 4.04)
	Viet Nam	100267854	41.45	1103731 (409581 to 2670612)	123.08	1100.78 (408.49 to 2663.48)	57.71	1118.13 (415.08 to 2700.97)	61.8	1.54 (1.52 to 1.56)
**Latin America and the Caribbean**										
	Antigua and Barbuda	89396	43.68	45 (5 to 123)	-64.1	50.52 (5.63 to 137.43)	-75.01	51.32 (5.71 to 139.61)	-74.39	-5.16 (-6.39 to -3.92)

Bahamas	387966	45.1	5307 (47 to 18325)	66.45	1367.82 (12.15 to 4723.48)	14.71	1375.07 (12.21 to 4748.53)	15.7	0.43 (0.23 to 0.64)
Barbados	299025	17.94	5764 (649 to 14315)	33.58	1927.45 (217.14 to 4787.21)	13.26	1933.88 (218.65 to 4805.35)	14.19	1.7 (1.56 to 1.84)
Belize	429075	118.74	2066 (199 to 6140)	198.64	481.55 (46.4 to 1431.1)	36.52	478.91 (46.13 to 1423.28)	38.37	2.63 (2.15 to 3.12)
Bermuda	63541	5.42	0 (0 to 0)	NA	0 (0 to 0)	NA	0 (0 to 0)	NA	-0.83 (-3.07 to 1.46)
Bolivia (Plurinational State of)	11797128	76.1	82854 (15421 to 183967)	208.04	702.32 (130.72 to 1559.42)	74.92	705.09 (131.06 to 1565.41)	76.56	2.65 (2.58 to 2.73)
Brazil	220356040	43.74	12863009 (3853876 to 26106042)	77.7	5837.38 (1748.93 to 11847.21)	23.63	5886.04 (1764.16 to 11932.19)	25.81	1.61 (1.59 to 1.62)
Colombia	49060426	44.46	737206 (182962 to 1464661)	106.46	1502.65 (372.93 to 2985.42)	42.92	1498.1 (372.27 to 2977.05)	43.03	2.95 (2.93 to 2.98)
Costa Rica	4747896	47.17	127337 (18843 to 315726)	57.59	2681.97 (396.88 to 6649.82)	7.08	2678.09 (394.95 to 6641.53)	7.8	1.28 (1.24 to 1.32)
Cuba	11269529	2.48	19075 (3735 to 51399)	45.32	169.26 (33.14 to 456.09)	41.8	169.39 (33.28 to 456.79)	41.21	1.15 (1.06 to 1.24)
Dominica	67077	-6.5	138 (12 to 404)	-45.03	205.78 (18.23 to 602.44)	-41.2	205.8 (18.22 to 602.47)	-40.71	0.2 (-0.63 to 1.04)
Dominican Republic	11012900	47.97	59174 (10394 to 138443)	129.12	537.32 (94.38 to 1257.1)	54.85	539.29 (94.77 to 1261.66)	56.39	2.76 (2.69 to 2.83)
Ecuador	18065389	72.47	94196 (22766 to 195368)	177.39	521.41 (126.02 to 1081.45)	60.84	521.48 (126.07 to 1082.87)	62.28	-0.17 (-0.23 to -0.12)
El Salvador	6450229	18.77	97130 (17377 to 222202)	74.83	1505.83 (269.4 to 3444.88)	47.2	1488.3 (266.18 to 3404.65)	47.31	2.26 (2.21 to 2.31)
Grenada	102627	16.17	344 (110 to 899)	404.64	335.4 (106.77 to 875.91)	334.4	338.83 (107.94 to 885.18)	347.4	5.23 (4.13 to 6.34)
Guatemala	15767372	77.63	36461 (7619 to 78848)	128.02	231.24 (48.32 to 500.07)	28.37	229.39 (47.87 to 495.98)	28.48	1.51 (1.4 to 1.62)
Guyana	764740	-1.81	1798 (158 to 5150)	27.69	235.09 (20.63 to 673.45)	30.04	236.52 (20.77 to 677.5)	30.99	2.53 (2.15 to 2.9)
Haiti	12864028	91.75	53436 (203 to 287598)	153.17	415.39 (1.58 to 2235.68)	32.03	417.09 (1.59 to 2244.8)	32.92	-0.41 (-0.5 to -0.32)
Honduras	10110156	102.75	89066 (8683 to 248182)	97.08	880.96 (85.89 to 2454.77)	-2.8	877.03 (85.72 to 2443.51)	-2.08	0.78 (0.72 to 0.85)
Jamaica	2799894	16.28	4352 (958 to 10035)	-23.89	155.43 (34.22 to 358.4)	-34.54	153.6 (33.87 to 354.18)	-34.16	-0.07 (-0.24 to 0.1)
Mexico	129275999	45.51	1403090 (1004122 to 1818973)	107	1085.34 (776.73 to 1407.05)	42.26	1088.49 (778.93 to 1410.87)	43.47	3.7 (3.69 to 3.72)
Nicaragua	6668976	61.67	75312 (28330 to 179689)	274.06	1129.28 (424.8 to 2694.41)	131.37	1129.92 (425.63 to 2678.84)	135.3	3.75 (3.66 to 3.84)
Panama	4292001	72.24	50264 (18240 to 142788)	254.56	1171.11 (424.98 to 3326.85)	105.84	1164.04 (422.32 to 3306.1)	106.52	2.07 (1.99 to 2.16)

Paraguay	7168841	67.55	180187 (48740 to 380402)	232.36	2513.47 (679.89 to 5306.32)	98.37	2506.64 (678.47 to 5302.03)	99.78	2.41 (2.36 to 2.46)
Peru	36270781	61.44	214658 (67022 to 534030)	239.39	591.82 (184.78 to 1472.34)	110.22	592.85 (185.1 to 1475.31)	112.9	3.58 (3.53 to 3.62)
Puerto Rico	3294345	-10.43	57087 (107 to 230222)	-18.47	1732.87 (3.25 to 6988.39)	-8.98	1667.65 (3.17 to 6725.48)	-12.36	-0.1 (-0.14 to -0.06)
Saint Kitts and Nevis	58645	40.75	39 (1 to 301)	10.63	67.2 (2.53 to 512.83)	-21.4	68.79 (2.59 to 524.95)	-18.46	-0.63 (-2.42 to 1.2)
Saint Lucia	177512	26.57	435 (99 to 1060)	127.73	245.28 (55.62 to 597.1)	79.92	248.35 (56.22 to 605.96)	84.42	3.07 (2.31 to 3.84)
Saint Vincent and the Grenadines	114075	3.77	385 (16 to 1236)	-38.01	337.58 (13.97 to 1083.75)	-40.26	339.61 (14.05 to 1090.24)	-38.76	-1.28 (-1.82 to -0.74)
Suriname	579247	48.62	1714 (35 to 5180)	23.67	295.93 (6.07 to 894.26)	-16.79	296.7 (6.09 to 896.6)	-15.68	-3.18 (-3.46 to -2.9)
Trinidad and Tobago	1393136	13.8	8068 (363 to 24300)	17.67	579.15 (26.07 to 1744.25)	3.4	583.17 (26.24 to 1756.41)	4.86	-0.04 (-0.17 to 0.09)
United States Virgin Islands	85902	-19.72	161 (3 to 917)	-36.49	186.91 (3.03 to 1068.05)	-20.88	187.33 (3.06 to 1070.51)	-21.74	-0.76 (-1.47 to -0.04)
Venezuela (Bolivarian Republic of)	26628840	35	270776 (34700 to 665509)	25.31	1016.85 (130.31 to 2499.21)	-7.18	1022.45 (131 to 2513.07)	-6.15	0.33 (0.3 to 0.35)

Notes:

All-age incidence rate: crude incidence rate.

Age-standardized incidence rate is computed by direct standardization with global standard population in GBD 2021.

Net drifts are estimates derived from the age-period-cohort model and denotes overall annual percentage change in age standardized incidence, which captures the contribution of the effects from calendar time and successive birth cohorts.

Parentheses for all GBD health estimate indicate 95% uncertainty intervals; parentheses for net drift indicate 95% confidence intervals.

APC: age-period-cohort.
